# The mitotic kinesin-14 KlpA contains a context-dependent directionality switch

**DOI:** 10.1038/ncomms13999

**Published:** 2017-01-04

**Authors:** Andrew R. Popchock, Kuo-Fu Tseng, Pan Wang, P. Andrew Karplus, Xin Xiang, Weihong Qiu

**Affiliations:** 1 Department of Biochemistry and Biophysics, Oregon State University, Corvallis, Oregon 97331, USA; 2 Department of Physics, Oregon State University, Corvallis, Oregon 97331, USA; 3 School of Physics and Electronics, Henan University, Kaifeng, Henan 475004, China; 4 Department of Biochemistry and Molecular Biology, The Uniformed Services University of the Health Sciences, Bethesda, Maryland 20814, USA

## Abstract

Kinesin-14s are commonly known as nonprocessive minus end-directed microtubule motors that function mainly for mitotic spindle assembly. Here we show using total internal reflection fluorescence microscopy that KlpA—a kinesin-14 from *Aspergillus nidulans*—is a context-dependent bidirectional motor. KlpA exhibits plus end-directed processive motility on single microtubules, but reverts to canonical minus end-directed motility when anchored on the surface in microtubule-gliding experiments or interacting with a pair of microtubules in microtubule-sliding experiments. Plus end-directed processive motility of KlpA on single microtubules depends on its N-terminal nonmotor microtubule-binding tail, as KlpA without the tail is nonprocessive and minus end-directed. We suggest that the tail is a *de facto* directionality switch for KlpA motility: when the tail binds to the same microtubule as the motor domain, KlpA is a plus end-directed processive motor; in contrast, when the tail detaches from the microtubule to which the motor domain binds, KlpA becomes minus end-directed.

## Abstract (web summary)

Kinesin-14s are commonly considered to be minus end-directed microtubule motor proteins. Here the authors show that KlpA, a fungal kinesin-14 orthologue, relies on its N-terminal nonmotor microtubule-binding tail to achieve context-dependent bidirectional motility.

Kinesins are microtubule motor proteins that convert the energy of ATP hydrolysis into mechanical work for various essential cellular processes^1^,^2^,^3^. The mitotic spindle is a microtubule-based bipolar machine in eukaryotes that separates duplicated chromosomes to ensure that daughter cells each receive proper genetic material during cell division^4^. Several different kinesin motor proteins are orchestrated inside the mitotic spindle for its assembly and maintenance^5^,^6^. Of all mitotic kinesins, kinesin-14s (that is, kinesins with a C-terminal motor domain) are commonly considered to be nonprocessive minus end-directed microtubule motors^7^,^8^,^9^,^10^,^11^,^12^,^13^,^14^,^15^,^16^. Loss of kinesin-14s has been shown to cause erroneous chromosome segregation^12^,^17^,^18^,^19^,^20^,^21^,^22^. In cancer cells, the human kinesin-14 HSET/KIFC1 is needed for clustering multiple centrosomes, a process crucial for cancer cell proliferation and survival^23^.

KlpA is a mitotic kinesin-14 from the filamentous fungus *Aspergillus nidulans*^24^. It is worth noting that *A. nidulans* is also the model organism for the discovery of BimC, the founding member of mitotic kinesin-5s (ref. 25). Like mitotic kinesin-14s in other eukaryotic cells^15^,^26^,^27^, KlpA counteracts the function of BimC^24^. Although KlpA is nonessential in wild-type cells^24^, its loss becomes synthetically lethal with gamma tubulin mutations^28^. KlpA is an attractive model protein for dissecting the mechanism and function of kinesin-14s, as its loss-of-function mutations can be conveniently isolated as suppressors of the bimC4 mutation^29^. However, compared with other mitotic kinesin-14s such as Ncd from *Drosophila melanogaster*, Pkl1 and Klp2 from *Schizosaccharomyces pombe*, and Kar3 from *Saccharomyces cerevisiae*, KlpA is much less well studied.

In this study, we report our *in vitro* characterization of KlpA motility in a variety of contexts using total internal reflection fluorescence (TIRF) microscopy. We find that, unlike all other kinesin-14s that have been studied to date, KlpA is a novel context-dependent bidirectional kinesin-14 motor: on single microtubules, KlpA unexpectedly moves towards the plus end in a processive manner, but when anchored on the coverslip (as in microtubule-gliding experiments) or interacting with a pair of microtubules (as in microtubule-sliding experiments), it reverts to exhibit canonical minus end-directed motility. We further show that KlpA requires its N-terminal nonmotor microtubule-binding tail (tail) for plus end-directed processive motility, as KlpA without this tail is minus end-directed in microtubule-gliding experiments and fails to generate processive motility on single microtubules. Collectively, these results indicate that the tail of KlpA plays a novel function as a switch for controlling its direction of motion in different contexts. This study sheds new insight into KlpA motor mechanisms and also markedly expands our knowledge of the diversified design principles of kinesin-14s.

## Results

### KlpA glides microtubules with minus end-directed motility

**Figure 1:**
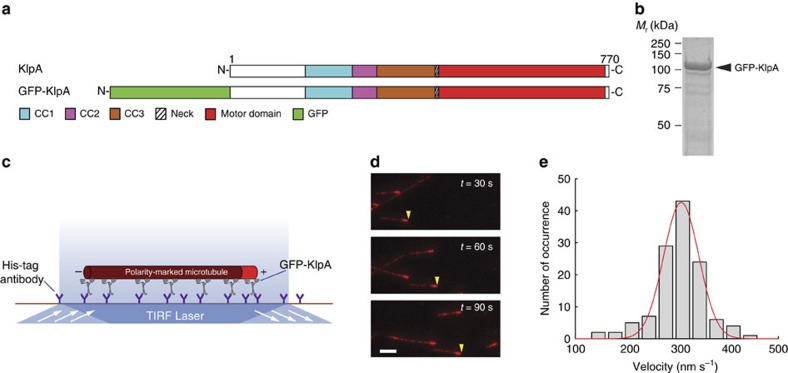
Surface-immobilized KlpA molecules exhibit minus end-directed motility to glide microtubules. (**a**) Schematic diagrams of the full-length KlpA and the recombinant GFP-KlpA. The full-length KlpA consists of three consecutive coiled coils (CC1, aa 153–249; CC2, aa 250–297; and CC3, aa 298–416), a neck (aa 417–421) and a catalytic microtubule-binding motor domain (aa 422–756). GFP-KlpA contains an N-terminal polyhistidine-tag (not shown). (**b**) Coomassie-stained SDS–polyacrylamide gel electrophoresis (SDS–PAGE) of purified recombinant GFP-KlpA. (**c**) Schematic diagram of the microtubule-gliding assay. Movement of microtubules driven by surface-immobilized GFP-KlpA molecules was visualized by TIRF microscopy. Microtubules were fluorescently labelled with tetramethylrhodamine (TMR), and polarity-marked with a dim minus end and a bright plus end. (**d**) Representative TIRF microscopy images of polarity-marked microtubules moving on the coverslip surface with the bright plus ends leading (yellow arrowheads). (**e**) Histogram showing the microtubule-gliding velocity distribution of GFP-KlpA. Red line indicates a Gaussian fit to the velocity histogram. Scale bar, 5 μm.

We set out to determine the directionality of KlpA *in vitro* using TIRF microscopy. To that end, we purified the recombinant full-length KlpA tagged with an N-terminal green fluorescent protein (GFP-KlpA, Fig. 1a,b). Because KlpA substitutes for Kar3 in *S. cerevisiae*^24^ and Kar3 forms a heterodimer with the nonmotor proteins Cik1 or Vik1(ref. 30), we performed two different assays—hydrodynamic analysis and single-molecule photobleaching—to determine the oligomerization status of KlpA. The hydrodynamic analysis yielded a molecular weight that is close to the theoretical value of a GFP-KlpA homodimer (Supplementary Fig. 1a,b). The photobleaching assay showed that the GFP fluorescence of GFP-KlpA was photobleached predominantly in a single step or two steps (Supplementary Fig. 1c,d), similar to other dimeric kinesins^31^. Thus, KlpA formed a homodimer in our *in vitro* experiments.

We next performed a microtubule-gliding assay to determine the directionality of KlpA (Fig. 1c). Briefly, GFP-KlpA molecules were immobilized on the coverslip via an N-terminal polyhistidine-tag, and KlpA directionality was deduced from the motion of polarity-marked microtubules. The assay showed that GFP-KlpA caused polarity-marked microtubules to move with the bright plus ends leading (Fig. 1d and Supplementary Movie 1) and a mean velocity of 309±35 nm s^−1^ (mean±s.d., *n*=123, Fig. 1e). In a control microtubule-gliding experiment using the plus end-directed human conventional kinesin hKHC^32^, microtubules were driven to move with the bright plus ends trailing (Supplementary Fig. 2 and Supplementary Movie 2). Taken together, these results demonstrate that KlpA, when anchored on the surface via its N terminus, is a minus end-directed motor protein, in agreement with a previous study using KlpA from clarified bacterial lysates^28^.

### KlpA is a plus end-directed processive kinesin-14 motor

**Figure 2:**
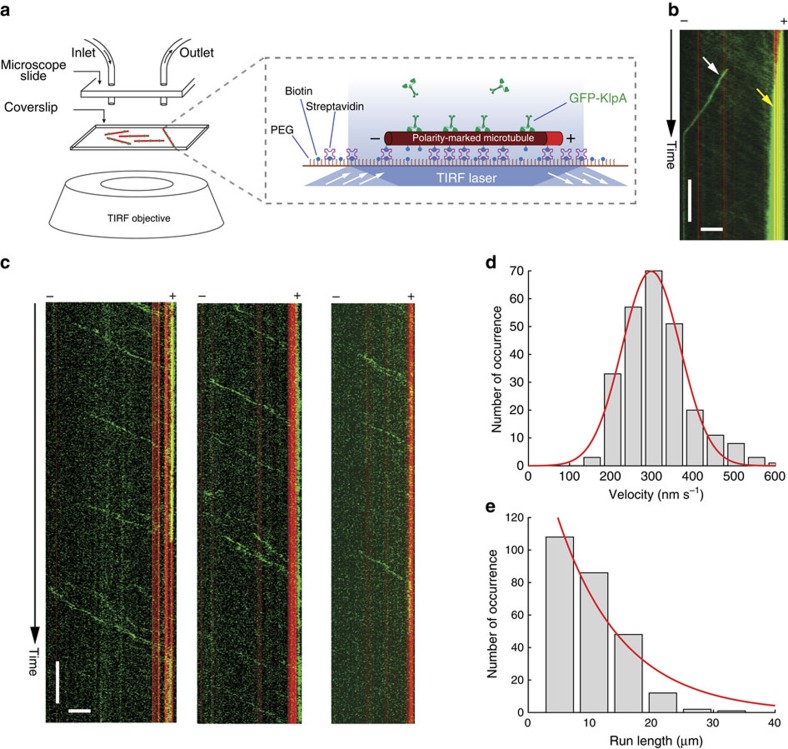
KlpA moves processively towards the plus end on single microtubules. (**a**) Schematic diagram of the *in vitro* KlpA motility assay. Microtubules were fluorescently labelled with Hilyte 647, and polarity-marked with a dim minus end and a bright plus end. (**b**) Example kymograph showing GFP-KlpA molecules (green), at relatively high protein input levels, form a plus end-directed flux and accumulate there on a surface-immobilized polarity-marked microtubule (red). Yellow arrow indicates GFP-KlpA accumulation at the microtubule plus end, and white arrow indicates minus end-directed movement of a GFP-KlpA aggregate. (**c**) Example kymographs showing that individual GFP-KlpA molecules (green) move towards the plus end on single polarity-marked microtubules (red) in a processive manner. (**d**) Velocity histogram of individual GFP-KlpA molecules on single microtubules. Red line indicates a Gaussian fit to the velocity histogram. (**e**) Run-length histogram of individual GFP-KlpA molecules on single microtubules. Red line indicates a single exponential fit to the run-length histogram. Scale bars, 1 min (vertical) and 5 μm (horizontal).

With the lone exception of Kar3, which moves processively on single microtubules towards the minus end by forming a heterodimer with the nonmotor proteins Cik1 or Vik1 (refs 30, 33), all other kinesin-14s that have been studied to date are exclusively nonprocessive minus end-directed motors. We thus wanted to determine whether KlpA is a typical kinesin-14 that lacks the ability to move processively on single microtubules as a homodimer. To address this, we performed an *in vitro* motility assay to visualize the movement of KlpA molecules on surface-immobilized polarity-marked microtubules (Fig. 2a). The assay showed that, contrary to the notion of kinesin-14s as minus end-directed motors, GFP-KlpA molecules unexpectedly formed a steady flux of plus end-directed motion and accumulated at the microtubule plus end (yellow arrow, Fig. 2b and Supplementary Movie 3). Occasionally, there were GFP-KlpA particles moving towards the microtubule minus ends (white arrow, Fig. 2b), but these minus end-directed particles were significantly brighter than the ones moving towards the plus end, implying that they were aggregates rather than simple homodimers.

Since the aforementioned motility experiments were performed at relatively high input levels of GFP-KlpA (≥4.5 nM; Fig. 2b), we repeated the same motility assay at much lower protein input levels (≤0.2 nM) so that the motile behaviour of individual GFP-KlpA molecules could be distinguished. The assay showed that individual GFP-KlpA molecules moved preferentially towards the microtubule plus end in a processive manner (Fig. 2c and Supplementary Movie 4) with a mean velocity of 300±70 nm s^−1^ (mean±s.d., *n*=257, Fig. 2d) and a characteristic run-length of 10.5±2.2 μm (mean±s.e.m., *n*=257, Fig. 2e). This run-length likely was an underestimate because most KlpA molecules reached the microtubule plus end. We also performed the mean square displacement (MSD) analysis, which showed that the motility of KlpA is dominated by directional movement (Supplementary Fig. 3). Together, these results demonstrate that, in direct contrast to all other kinesin-14s that have been analysed to date, KlpA is a novel kinesin-14 motor that uniquely exhibits plus end-directed processive motility on single microtubules. It is worth emphasizing that such novel processive motility is not due to coupling^31^,^34^, but rather an intrinsic behaviour of a single KlpA homodimer.

### Plus end-directed KlpA motility requires its N-terminal tail

**Figure 3:**
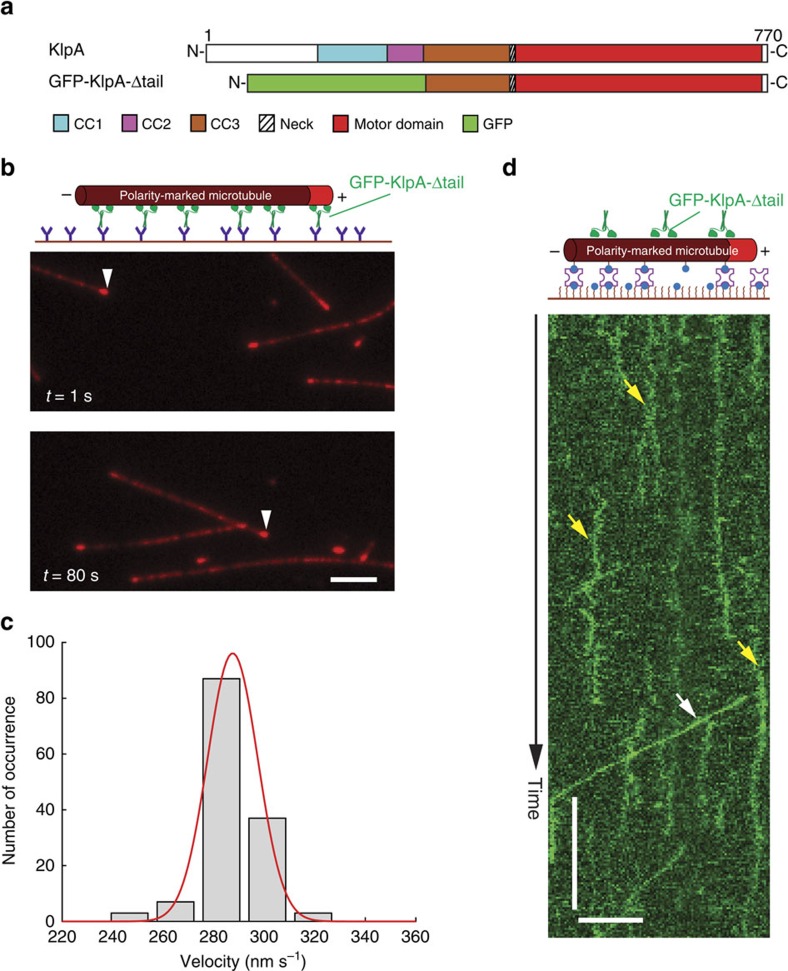
Plus end-directed processive motility of KlpA on single microtubules requires its N-terminal nonmotor microtubule-binding tail. (**a**) Schematic diagrams of the full-length KlpA and the recombinant GFP-KlpA-Δtail. GFP-KlpA-Δtail contains a polyhistidine-tag (not shown) and a GFP at the N terminus, and residues 303–770 of KlpA. (**b**) Representative TIRF microscopy images of GFP-KlpA-Δtail driving polarity-marked microtubules (red) to glide with the bright plus ends leading (white arrowheads). (**c**) Velocity histogram of microtubule-gliding by GFP-KlpA-Δtail. Red line indicates a Gaussian fit to the velocity histogram. (**d**) Example kymograph showing that individual GFP-KlpA-Δtail molecules (yellow) exhibit nonprocessive movement on a single polarity-marked microtubule with a bright plus end. White arrow indicates minus end-directed movement of a rare GFP-KlpA-Δtail aggregate. Scale bars, 1 min (vertical) and 5 μm (horizontal).

Like other kinesin-14s such as Klp2 in *S. pombe* and Ncd in *D. melanogaster*^35^,^36^, KlpA was also able to slide apart antiparallel microtubules and to statically crosslink parallel microtubules via its N-terminal nonmotor microtubule-binding tail (Supplementary Fig. 4a–g and Supplementary Movies 5 and 6). As several other kinesins are known to rely on nonmotor microtubule-binding domains to either achieve processive motility^30^ or enhance processivity^37^,^38^, we sought to determine whether the microtubule-binding tail of KlpA plays a similar role in enabling its unexpected plus end-directed processive motility on single microtubules. To do this, we purified GFP-KlpA-Δtail (Fig. 3a), a truncated construct lacking the N-terminal tail, for *in vitro* motility experiments. Like GFP-KlpA, GFP-KlpA-Δtail formed a homodimer (Supplementary Fig. 5), and exhibited minus end-directed motility in the microtubule-gliding assay (Fig. 3b and Supplementary Movie 7) with a mean velocity of 287±10 nm s^−1^ (mean±s.d., *n*=138, Fig. 3c). This latter observation implies that motor–neck core of KlpA is inherently minus end-directed, as would be expected based on its highly conserved neck^32^,^39^,^40^,^41^. However, the *in vitro* motility assay showed that GFP-KlpA-Δtail did not form a steady flux towards either end of the microtubule, nor did it accumulate at the microtubule ends (Fig. 3d and Supplementary Movie 8). Although some occasional brighter and presumably aggregated particles moved processively towards the microtubule minus ends (white arrow, Fig. 3d and Supplementary Movie 8), individual GFP-KlpA-Δtail molecules behaved like other nonprocessive kinesin-14s (refs 31, 36, 42) and mostly interacted with the microtubules in a diffusive manner with no apparent directional preference (yellow arrow, Fig. 3d and Supplementary Movie 8). The MSD analysis showed that the motility of GFP-KlpA-Δtail on single microtubules is best described by one-dimensional diffusion (Supplementary Fig. 6). Thus, besides allowing for microtubule-sliding and crosslinking, the tail of KlpA has an additional novel functionality of enabling the kinesin-14 motor to move on single microtubules towards the plus end in a processive manner.

### KlpA exhibits context-dependent directional preferences

**Figure 4:**
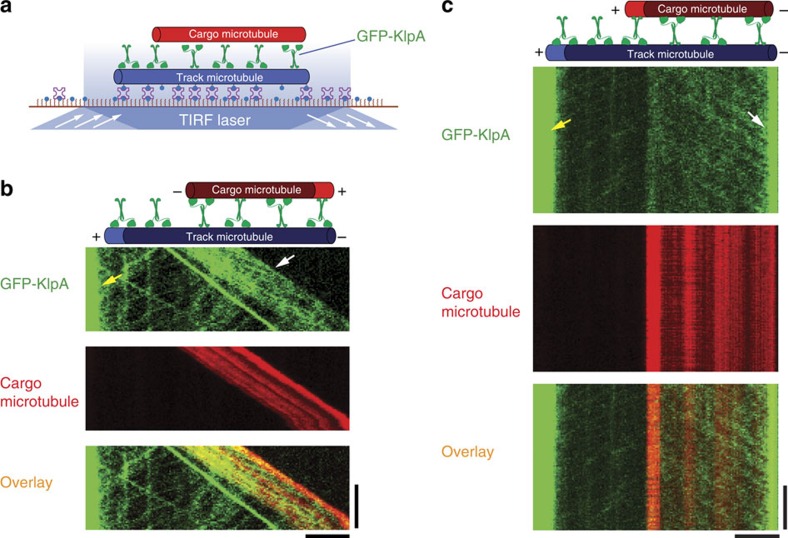
KlpA exhibits opposite directional preference inside and outside the microtubule overlaps. (**a**) Schematic diagram of the microtubule-sliding assay showing that KlpA contains context-dependent opposite directional preference. Track and cargo microtubules were fluorescently labelled with Hilyte 647 and TMR, respectively, and polarity-marked with a dim minus end and a bright plus end. (**b**) Example kymographs of GFP-KlpA motility inside and outside the antiparallel microtubule overlap. Yellow arrow indicates GFP-KlpA accumulation at the microtubule plus end outside the antiparallel microtubule overlap. White arrow indicates minus end-directed movement of GFP-KlpA inside the antiparallel microtubule overlap. (**c**) Example kymographs of GFP-KlpA motility inside and outside the parallel microtubule overlap. Yellow arrow indicates GFP-KlpA accumulation at the microtubule plus end outside the parallel microtubule overlap. White arrow indicates GFP-KlpA accumulation at the microtubule minus end inside the parallel microtubule overlap. Scale bars, 30 s (vertical) and 5 μm (horizontal).

From the opposite directional preference exhibited by KlpA in the ensemble microtubule assays (Fig. 1d and Supplementary Fig. 4c) and the single-molecule motility experiments (Fig. 2b,c), we inferred that KlpA contains a context-dependent mechanism to switch directions on the microtubule. We thus directly compared the motility of GFP-KlpA inside and outside the microtubule overlap on the same track microtubule using a microtubule-sliding assay (Fig. 4a), as has been done previously for *S. cerevisiae* kinesin-5 Cin8 (ref. 43). Briefly, in this assay the track (blue) and cargo (red) microtubules were both polarity-marked but labelled with different dyes; track microtubules were first immobilized on a coverslip inside the motility chamber and bound with purified GFP-KlpA molecules; moreover, cargo microtubules were added into the chamber before three-colour time-lapse imaging was acquired to simultaneously visualize the motility of GFP-KlpA molecules and cargo microtubules on the same track microtubules. Like KlpA, GFP-KlpA was also able to slide antiparallel microtubules relative to each other (Fig. 4b) and to statically crosslink parallel microtubules (Fig. 4c). In both scenarios, when outside the microtubule overlap regions, GFP-KlpA molecules showed a plus end-directed flux and accumulated at the plus end on the track microtubule (yellow arrow, Fig. 4b,c and Supplementary Movies 9 and 10). This matched the behaviour of GFP-KlpA on single microtubules (Fig. 2b). In contrast, inside the antiparallel microtubule overlap regions, GFP-KlpA molecules carried the cargo microtubule towards the minus end of the track microtubule (white arrow, Fig. 4b and Supplementary Movie 9). In the parallel orientation, the cargo microtubule remained stationary on the track microtubule, but GFP-KlpA molecules moved preferentially towards and gradually accumulated at the minus end inside the parallel microtubule overlap (white arrow, Fig. 4c and Supplementary Movie 10). This is similar to the observation that Ncd preferentially accumulates at the minus ends between statically crosslinked parallel microtubules^36^. Collectively, these results demonstrate that KlpA can, depending on its context, display opposite directional preferences on the same microtubule: it is plus end-directed outside the microtubule overlap regions and minus end-directed inside the microtubule overlap regions regardless of the relative microtubule polarity.

## Discussion

Kinesin-14 has been an intriguing kinesin subfamily since the discovery of its founding member Ncd^44^,^45^ because all kinesin-14s studied to date are exclusively minus end-directed based on the microtubule-gliding experiments^31^,^35^,^42^,^44^,^45^,^46^,^47^,^48^,^49^. In addition, no kinesin-14 has been shown to be able to generate processive motility on the microtubule as a single homodimer. Kar3 is the only kinesin-14 known to generate minus end-directed processive motility on single microtubules without clustering, and it does that by forming a heterodimer with its associated light chains Vik1 or Cik1 (refs 30, 33). To our knowledge, KlpA is the first kinesin-14 that exhibits both plus end-directed processive motility on single microtubules and context-dependent directional switching. Thus, our study markedly expands the diversity of kinesin-14s.

Our results show that while the full-length KlpA clearly moves towards the plus end on single microtubules in a processive manner (Fig. 2b,c), a truncated KlpA lacking the N-terminal microtubule-binding tail glides microtubules with minus end-directed motility (Fig. 3b) but becomes nonprocessive on single microtubules (Fig. 3d). There are several important implications from these observations. First, without the tail, the motor–neck core of KlpA is inherently minus end-directed, which is consistent with the notion that all kinesin-14s share a highly conserved neck that serves as the minus end directionality determinant^32^,^39^,^40^,^41^. Second, on single microtubules, the tail enables KlpA to exhibit both processive motility and plus end directionality. We propose that on a single microtubule, KlpA assumes a *cis* conformation in which the catalytic microtubule-binding motor domain and the tail bind to the same microtubule. Binding of the tail to the *cis*-microtubule subsequently prevents KlpA from premature dissociation, and this enables the kinesin to move processively on the microtubule, as has been observed previously for Kar3, which depends on the nonmotor microtubule-binding domain in Vik1 or Cik1 for processive motility on microtubules^30^.

At present, it is unclear how exactly the tail enables the full-length KlpA to move preferentially towards the plus end on a single microtubule at the atomic level. Previous studies^50^,^51^,^52^ suggest that kinesin-14 motors use a lever mechanism for minus end-directed motility, where the neck acts as an extended lever arm and undergoes rotation towards the microtubule minus end upon ATP binding. Building on this, we speculate that when KlpA adopts the *cis* conformation on a single microtubule, binding of the tail on the surface of the same microtubule as the motor domain induces a conformational strain through the stalk that causes the neck to rotate towards the microtubule plus end, thereby enabling the kinesin for plus end-directed motility. The precise underlying mechanism of plus end-directed KlpA processivity awaits future single-molecule studies of designed KlpA variants as well as cryo-electron microscopy (cryo-EM) studies of these variants on single microtubules.

Among all the experiments, KlpA exhibits plus end-directed motility only on single microtubules (Fig. 2b,c); moreover, in this scenario, the tail of KlpA and its catalytic motor domain are expected to bind to the same microtubule. In contrast, KlpA exhibits canonical minus end-directed motility when it is either anchored on the coverslip via the N terminus in the ensemble microtubule-gliding experiments (Fig. 1c,d) or between a pair of microtubules in the microtubule-sliding experiments (Fig. 4b,c). In both scenarios, the N-terminal microtubule-binding tail of KlpA is not attached to the microtubule to which its motor domain binds. These results suggest that the tail of KlpA is a *de facto* directionality switch: to achieve plus end-directed processive motility, the switch-like tail of KlpA must bind to the same microtubule as its catalytic motor domain; moreover, to achieve minus end-directed motility, the switch needs to be detached from the microtubule to which its motor domain binds.

Our results show that KlpA accumulates at the plus end on single microtubules (Fig. 2b,c), and we suggest that this is enabled by its tail via a mechanism similar to that of the budding yeast kinesin-8 Kip3 (ref. 53). The tail likely binds strongly to the splayed protofilaments at the microtubule plus end to enhance the retention of KlpA there. It is worth noting that such accumulation requires the motor activity of KlpA to reach the microtubule plus end, as purified tail does not accumulate at either end of the microtubule and instead decorates the entire length of the microtubule rather uniformly (Supplementary Fig. 4e).

It has long been established that KlpA counteracts the activity of BimC^24^, but the underlying mechanism has been largely unknown. How could our results relate to the *in vivo* functions of KlpA? While KlpA localization inside the mitotic spindle has not yet been studied, one potential site of localization is the spindle pole, as several mitotic kinesin-14s, including HSET, CHO2, Kar3, Ncd, XCTK2 and Pkl1, are all known to localize to the spindle poles^54^,^55^,^56^. If KlpA does localize to the spindle poles, then how does it localize there? A recent study has shown that that Pkl1—a mitotic kinesin-14 from fission yeast—forms a complex with Msd1 and Wdr8 for translocating to and anchoring at the spindle poles^55^. As homologues of Msd1 and Wdr8 are both present in *A. nidulans*^57^, it is plausible that binding of Msd1 and/or Wdr8-like proteins to KlpA dislodges its N-terminal tail from the *cis*-microtubule surface, and this activates the kinesin for minus end-directed motility both on single microtubules and at the spindle poles. The spindle midzone is another potential site of localization. In this case, our results suggest that while other mitotic kinesin-14s appear to depend on partner proteins to localize to the microtubule plus end^14^,^47^,^58^,^59^, KlpA can in principle autonomously localize to the spindle midzone via its inherent plus end-directed processive motility.

Several mitotic kinesin-5s were recently shown to be context-dependent bidirectional motor proteins^43^,^60^,^61^,^62^, indicating that context-dependent directional switching is evolutionarily conserved among kinesin-5s. Our current work on KlpA provides the first evidence to suggest that context-dependent directional switching could also exist among some, if not all, mitotic kinesin-14s. Thus, the mechanism and regulation of bidirectional mitotic kinesins will be an important subject for future studies.

## Methods

### Molecular cloning of recombinant KlpA constructs

The full-length cDNA of KlpA was codon-optimized and synthesized for enhanced protein expression in bacteria. All recombinant KlpA constructs were integrated in a modified pET-17b vector (Novagen) using either isothermal assembly or the Q5 site-directed mutagenesis kit (NEB) and verified by DNA sequencing. All KlpA constructs contained an N-terminal 6 × His-tag for protein purification. Similarly, the recombinant human conventional kinesin hKHC(1–560) with a C-terminal HaloTag (Promega) and 6xHis-tag was derived from a gift plasmid from the Reck-Peterson laboratory at UCSD.

### Protein expression and purification

All protein constructs were expressed in BL21(DE3) Rosetta cells (Novagen). Cells were grown at 37 °C in tryptone phosphate medium (TPM) supplemented with 50 μg ml^−1^ Ampicillin and 30 μg ml^−1^ chloramphenicol until OD_600_=0.8. Expression was induced with 0.1 mM isopropyl-β-D-thiogalactoside for 12–14 h at 20 °C. Cells were harvested by centrifugation, flash-frozen in liquid nitrogen and stored at −80 °C.

For protein purification, cell pellets were resuspended in 50 mM sodium phosphate (NaP_i_) buffer (pH 8.0) containing 250 mM NaCl, 1 mM MgCl_2_, 0.5 mM ATP, 10 mM β-mercaptoethanol, 5% glycerol and 20 mM imidazole in the presence of a protease inhibitor cocktail and then lysed via sonication. After centrifugation, soluble protein in the supernatant was purified by Talon resin (Clontech) and eluted into 50 mM NaP_i_ buffer (pH 7.2) containing 250 mM NaCl, 1 mM MgCl_2_, 0.5 mM ATP, 10 mM β-mercaptoethanol, 5% glycerol and 250 mM imidazole. Protein was then flash-frozen in liquid nitrogen and stored at −80 °C.

### Hydrodynamic analysis

To determine the size of a recombinant KlpA construct, a combination of gel filtration and sucrose gradient centrifugation was used as described previously^63^. For gel filtration, 500 μl of purified protein was applied to a Superdex 200 (GE Life Sciences) column pre-equilibrated with BRB50 (50 mM PIPES, pH 6.8, 1 mM EGTA and 1 mM MgCl_2_) supplemented with 100 mM KCl. *A*_280_ was monitored as 500-μl fractions were collected followed by SDS–PAGE analysis. Fraction intensity was calculated using ImageJ (NIH) and elution peak was determined with Origin 7.0 (OriginLab). For standard proteins, 300 μl of solution containing each standard at 3 mg ml^−1^ was applied to the column and analysed similarly. The Stokes radius of a given KlpA construct was determined using the plot of the Stokes radius of standard proteins (Thyroglobulin, 8.5 nm; β-amylase, 5.4 nm; BSA, 3.55 nm) versus peak elution volume.

For sucrose gradient centrifugation, 100 μl of purified protein was applied to an 11 ml 5–20% (w/v) sucrose gradient in BRB50 supplemented with 100 mM KCl buffer. The gradient was centrifuged in an SW41 rotor at 150,000 *g* for 18 h at 4 °C. Fractions (420 μl) were collected from the top of the gradient and analysed via SDS–PAGE. For standard proteins, 50 μl of solution containing each standard at 3 mg ml^−1^ was applied to the same gradient. Fraction intensity was calculated using ImageJ (NIH) and elution peak was determined with Origin 7.0 (OriginLab). The sedimentation coefficient was determined by plotting the peak elution fraction versus the sedimentation coefficient of standard proteins (alcohol dehydrogenase, 7.4 S; BSA, 4.4 S; carbonic anhydrase, 2.8 S).

### Preparation of polarity-marked microtubules

All taxol-stabilized polarity-marked microtubules (tetramethylrhodamine (TMR), Alexa 488, and HiLyte 647) with bright plus ends were prepared as previously described^64^. To make the polarity-marked microtubules, a dim tubulin mix (containing 17 μM unlabelled tubulin and 0.8 μM fluorescently labelled tubulin) was first incubated in BRB80 (80 mM PIPES, pH 6.8, 1 mM EGTA and 1 mM MgCl_2_) with 0.5 mM guanosine-5′-[(α,β)-methyleno]triphosphate (GMPCPP) (Jena Bioscience) at 37 °C for 2 h to make dim microtubules, and then centrifuged at 250,000 *g* for 7 min at 37 °C in a TLA100 rotor (Beckman). The pellet was resuspended in a bright tubulin mix (containing 7.5 μM unlabelled tubulin, 4 μM fluorescently labelled tubulin and 15 μM N-ethylmaleimide-tubulin) in BRB80 with 0.2 mM GMPCPP and incubated at 37 °C for additional 15 min to cap the plus ends. The resulting polarity-marked microtubules were pelleted at 20,000 *g* for 7 min at 37 °C in the TLA100 rotor and finally resuspended in BRB80 with 40 μM taxol. For making track microtubules used in single-molecule assays and microtubule-sliding assays, the dim tubulin mix also included additional 17 μM biotinylated tubulin.

### TIRF microscopy

All time-lapse imaging experiments were performed at room temperature using the Axio Observer Z1 objective-type TIRF microscope (Zeiss) equipped with a × 100 1.46 numerical aperture oil-immersion objective and a back-thinned electron multiplier charge-coupled device camera (Photometrics). Except for the microtubule-gliding experiments, which used regular coverslips, all other experiments used microscope coverslips that were functionalized with biotinylated polyethylene glycol (biotin-PEG) as previously described^65^ to reduce nonspecific surface absorption of molecules. All time-lapse imaging experiments in this study used flow chambers that were made by attaching a coverslip to a microscope glass slide by double-sided tape.

### Single-molecule photobleaching assays

For the single-molecule photobleaching assays, GFP-tagged KlpA motors were diluted and bound to surface-immobilized HyLite 647 microtubules in a BRB50-based buffer supplemented with 25 mM KCl, 20 μM taxol and 1.3 mg ml^−1^ casein, and 1.5 mM AMPPNP. To remove GFP contamination for the assay, the full-length GFP-KlpA was additionally purified via gel filtration in 50 mM NaP_i_ buffer (pH 7.2) containing 250 mM NaCl, 1 mM MgCl_2_, 0.5 mM ATP and 10 mM β-mercaptoethanol. Time-lapse images were continuously recorded with 200-ms exposure until the field of view was bleached. The number of photobleaching steps of individual KlpA motors was obtained by tracking the fluorescence intensity in ImageJ (NIH).

### I*n vitro* motility assays

For all *in vitro* motility experiments, the motility chamber was perfused with 0.5 mg ml^−1^ streptavidin for immobilizing polarity-marked HyLite 647 microtubules. GFP-tagged kinesin molecules (GFP-KlpA and GFP-KlpA-Δtail) were then diluted in motility buffer (BRB50 supplemented with 25 mM KCl, 1 mM ATP, 25 μM taxol, 1.3 mg ml^−1^ casein and an oxygen scavenger system^66^) and added to the chamber. Time-lapse images were acquired at 1 frame per second. The setting was 100-ms exposure and 5-min duration for the high-concentration flux experiments and 200-ms exposure and 10 min for single-molecule experiments to determine the velocity and run-length of GFP-KlpA. Kymographs were generated and analysed in ImageJ (NIH) for determining directionality, velocity and run-length information of GFP-tagged kinesin motors. Velocity and run-length were determined by fitting the histograms to a Gaussian distribution and an exponential distribution, respectively, in Matlab (MathWorks)

### Microtubule-gliding assays

For microtubule-gliding assays, kinesin motors (diluted in BRB50 supplemented with 20 μM taxol and 1.3 mg ml^−1^ casein) were immobilized on the coverslip via the monoclonal Anti-His antibody (Fisher Scientific). Unbound kinesin molecules were removed with BRB50 supplemented with 20 μM taxol and 1.3 mg ml^−1^ casein. Polarity-marked TMR microtubules diluted in the same buffer were then added to the chamber and incubated for 2 min. Unbound microtubules were removed by extensive wash with BRB50 supplemented with 20 μM taxol and 1.3 mg ml^−1^ casein. Finally, the chamber was perfused with a BRB50-based motility buffer supplemented with 100 mM KCl, 1 mM ATP, 25 μM taxol, 1.3 mg ml^−1^ casein and an oxygen scavenger system^66^. Time-lapse images were taken at 1 frame per second for 5 min.

### Microtubule-sliding assays

For all microtubule-sliding assays, the motility chamber was first perfused with 0.5 mg ml^−1^ streptavidin for immobilizing polarity-marked track microtubules. After washing out free track microtubules, the kinesin motor was diluted into BRB50 supplemented with 20 μM taxol and 1.3 mg ml^−1^ casein and added to the chamber and incubated to allow for binding to the track microtubules. All unbound motors were washed away before the addition of polarity-marked cargo microtubules. After incubation all non-bundled cargo microtubules were washed away with BRB50 supplemented with 20 μM taxol and 1.3 mg ml^−1^ casein before the addition of motility buffer (BRB50 supplemented with 25 mM KCl, 1 mM ATP, 25 μM taxol, 1.3 mg ml^−1^ casein and an oxygen scavenger system^66^). Time-lapse images were taken at 1 frame per second.

### MSD analysis

To perform the MSD analysis, single-molecule motility data of KlpA on polarity-marked microtubules were acquired with 100-ms exposure time and 130-ms interval for a total duration of 2 min. Sub-pixel *xy* coordinates of motile KlpA molecules were determined using TrackMate (http://fiji.sc/TrackMate). The MSD values were computed from these *xy* coordinates using the formula as previously described^67^. The MSD-versus-time plots were analysed by fitting curves to data using nonlinear regression in Matlab (MathWorks).

### Other microtubule-based assays

To confirm the microtubule-binding ability of KlpA-tail-GFP, HyLite 647 microtubules were immobilized on the surface of coverslip, and purified KlpA-tail-GFP diluted in BRB50 supplemented with 25 mM KCl, 25 μM taxol and 1.3 mg ml^−1^ casein was added in the flow chamber. Unbound KlpA-tail-GFP was washed away with BRB50 supplemented with 25 mM KCl, 25 μM taxol and 1.3 mg ml^−1^ casein after 2-min incubation, and TIRF microscopy images were taken for both HyLite 647 and GFP channels.

To confirm that the N-terminal tail is required for KlpA to crosslink microtubules, GFP-KlpA and GFP-KlpA-Δtail were each mixed with both HyLite 647 microtubules and TMR microtubules in BRB50 supplemented with 25 mM KCl, 25 μM taxol and 1.3 mg ml^−1^ casein, and added to the flow chamber after 2-min incubation for TIRF microscopy imaging in both HyLite 647 and TMR channels.

### Data availability

The data that support the findings of this study are available from the corresponding author upon reasonable request.

## Additional information

**How to cite this article:** Popchock, A. R. *et al*. The mitotic kinesin-14 KlpA contains a context-dependent directionality switch. *Nat. Commun.* **8,** 13999 doi: 10.1038/ncomms13999 (2017).

**Publisher's note:** Springer Nature remains neutral with regard to jurisdictional claims in published maps and institutional affiliations.

## Supplementary Material

Supplementary Information Supplementary Figures

Supplementary Movie 1 KlpA exhibits minus end-directed motility in the microtubule-gliding experiments. Movie showing that surface-immobilized GFP-KlpA molecules collectively drive polarity-marked microtubules (red) to glide with the bright plus ends leading. Microtubules are labeled with TMR. This movie corresponds to Fig. 1d.

Supplementary Movie 2 Purified human conventional kinesin heavy chain hKHC(1-560) exhibits plus end-directed motility in the microtubule-gliding experiments. Movie showing that surface-immobilized hKHC(1-560) collectively drive polarity-marked microtubules (red) to glide with the bright plus ends trailing. Microtubules are labeled TMR. This movie corresponds to Supplementary Fig. 2b.

Supplementary Movie 3 KlpA forms a steady flux toward and accumulates at the plus end on single-microtubules. Movie showing that GFP-KlpA molecules (green) form a plus end-directed flux and accumulate at the microtubule plus ends on surface immobilized polarity-marked Hilyte 647-microtubules (red) with a bright plus end. Top: the microtubule channel; Middle: the GFP-KlpA channel; Bottom: the overlay of the microtubule and GFP-KlpA channel. This movie corresponds to Fig 2b. Arrowhead indicates the plus end of the polarity microtubule used for generating the kymograph in Fig. 2b.

Supplementary Movie 4 KlpA moves processively toward the plus end on single microtubules. Movie showing that individual KlpA molecules (green) move processively toward the plus ends on surface-immobilized microtubules (red). Microtubules are fluorescently labeled with Hilyte 647 and polarity-marked with a bright plus end. Top: the microtubule channel; Middle: the GFP-KlpA channel; Bottom: the overlay of the microtubule and GFP-KlpA channel. The movie corresponds to Fig. 2c. Arrowhead indicates the plus end of the polarity microtubule used for generating the leftmost kymograph in Fig. 2c.

Supplementary Movie 5 KlpA slides antiparallel microtubules relative to each other with minus end-directed motility. Movie showing that, in the antiparallel orientation, KlpA molecules (unlabeled) collectively slide the cargo microtubules (green) on a surface-immobilized track microtubule (red) with minus end-directed motility. The cargo and track microtubule are both polarity-marked with a bright plus end and fluorescently labeled with Alex 488 and TMR respectively. This movie corresponds to Supplementary Fig. 4c.

Supplementary Movie 6 KlpA statically crosslinks parallel microtubules. Movie showing that the cargo microtubule (green) remains stationary on the surface-immobilized track microtubule (red), when induced to align in the parallel orientation relative to the track microtubule by KlpA molecules (unlabeled). The cargo and track microtubule are both polarity-marked with a bright plus end and fluorescently labeled with Alex 488 and TMR respectively. This movie corresponds to Supplementary Fig. 4d.

Supplementary Movie 7 KlpA-Δtail exhibits minus end-directed motility in the microtubule-gliding experiments. Movie showing that surface-immobilized GFP-KlpA-Δtail molecules via the N-terminal polyhistidine-tag collectively drive polarity-marked microtubules (red) to glide with the bright plus ends leading. Microtubules are labeled with TMR. This movie corresponds to Fig. 3b.

Supplementary Movie 8 GFP-KlpA-Δtail molecules are unable to form a plus end-directed flux and diffuse on single microtubules. Movie showing that GFP-KlpA-Δtail molecules (green) diffuse on surface-immobilized microtubules (red). Microtubules are fluorescently labeled with Hilyte 647 and polarity-marked with a bright plus end. Top: the microtubule channel; Middle: the GFP-KlpA-Δtail channel; Bottom: the overlay of the microtubule and GFP-KlpA-Δtail channel. Occasional processive minus end-directed particles are likely aggregates but not individual dimers based on their relative brightness. This movie corresponds to Fig. 3d. Arrowhead indicates the plus end of the polarity microtubule used for generating the kymograph in Fig. 3d.

Supplementary Movie 9 KlpA exhibits opposite directional preference inside and outside the antiparallel microtubule overlap. Movies showing that GFP-KlpA molecules (green) move preferentially toward the plus end of the track microtubule (blue) outside the antiparallel overlap and collectively transport the cargo microtubule (red) toward the minus end of the track microtubule. The cargo and track microtubule were both polarity-marked with a bright plus end, and fluorescently labeled with TMR and Hilyte 647 respectively. From Top to Bottom: the track microtubule channel (the movie was made from a single snapshot of the track microtubule); the GFP-KlpA channel; the cargo microtubule channel; and the overlay of the GFP-KlpA and cargo microtubule channels. Movie corresponds to Fig. 4b. Arrowhead indicates the plus end of the track microtubule used for generating the kymograph in Fig. 4b.

Supplementary Movie 10 KlpA exhibits opposite directional preference inside and outside the parallel microtubule overlap. Movies showing that GFP-KlpA molecules (green) move preferentially toward the plus end of the track microtubule (blue) outside the parallel overlap area but preferentially move to accumulate at the minus end of the cargo microtubule inside the parallel overlap area. The cargo and track microtubule were both polarity-marked with a bright plus end, and fluorescently labeled with TMR and Hilyte 647 respectively. Top: the track microtubule channel, and the movie was made from a single snapshot of the track microtubule; Second from the top: the GFP-KlpA channel; Third from the top: the cargo microtubule channel; Bottom: Overlay of the GFP-KlpA and cargo microtubule channels. Movie corresponds to Fig. 4c. Arrowhead indicates the plus end of the track microtubule used for generating the kymograph in Fig. 4c.
