# Supplementary material for: The mitotic kinesin-14 KlpA contains a context-dependent directionality switch
Source: Nat Commun. 2017 Jan 4;8:13999. doi: 10.1038/ncomms13999 (PMC5216134; doi:10.1038/ncomms13999)
Supplement: Supplementary Information — Supplementary Figures [file ncomms13999-s1.pdf]

**Supplementary Figures 1-6**

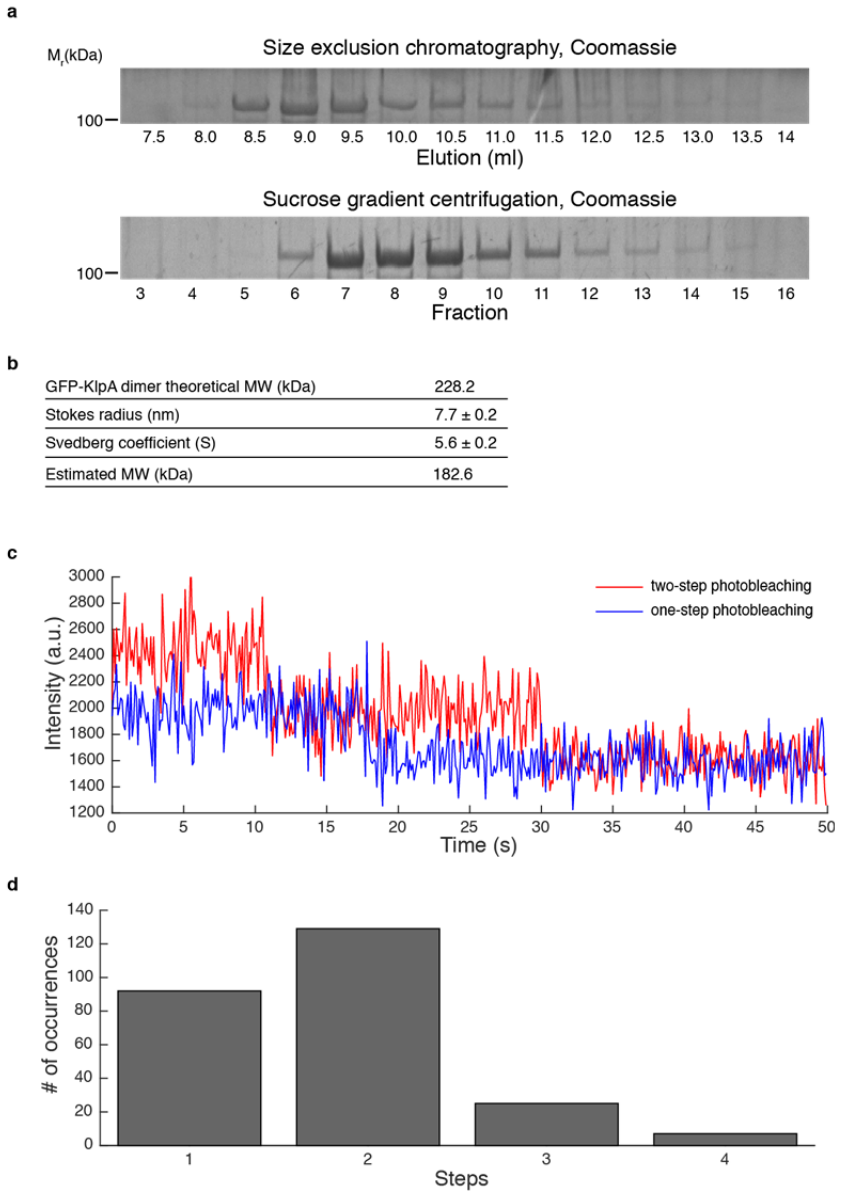


**Supplementary Fig. 1: GFP-KlpA forms a homodimer. a,** Hydrodynamic analysis of the purified full-length GFP-KlpA protein. Fractions from size exclusion chromatography and 5-20% (w v$^{-1}$) sucrose gradient centrifugation experiments. **b,** Calculation of GFP-KlpA molecular weight based on the experimentally derived Stokes radius and Svedberg coefficient. **c,** Representative photobleaching traces of GFP-KlpA fluorescence on surface-immobilized

microtubules in the presence of 1.5 mM AMPPNP. **d,** Histogram of fluorescence photobleaching steps of GFP-KlpA (n = 253).

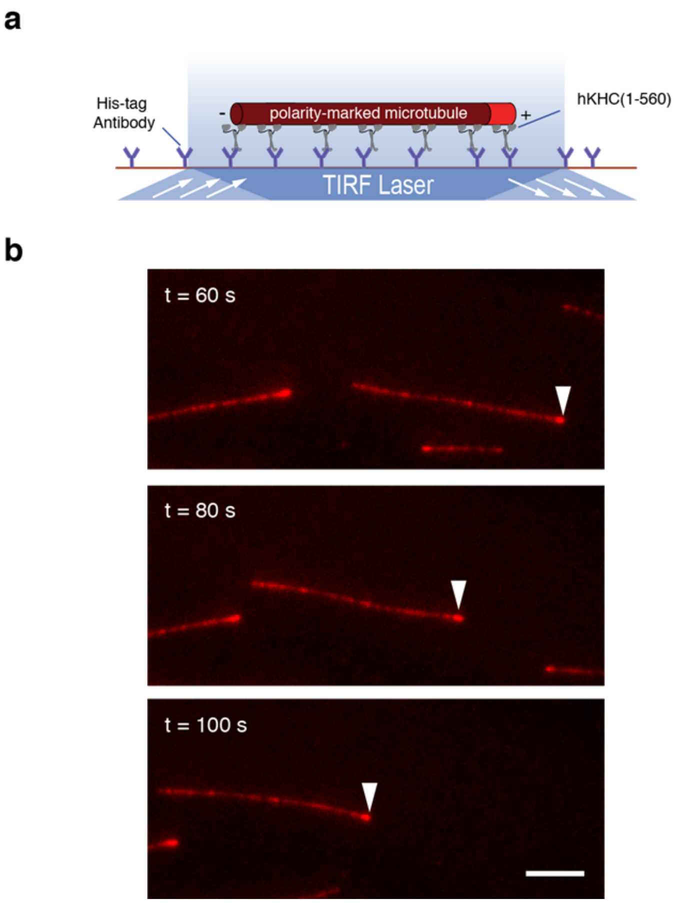


**Supplementary Fig. 2: Surface-immobilized human conventional kinesin hKHC(1-560) drives microtubule gliding with plus end-directed motility. a,** Schematic diagram of the microtubule-gliding assay. **b,** Representative TIRF microscopy images showing that surface-immobilized plus end-directed hKHC(1-560) molecules collectively drive microtubules to glide with the bright plus ends trailing. Microtubules are fluorescently labeled with TMR and polarity-marked with a dimly labeled fluorescent segment at the minus end and a more brightly labeled segment at the plus end. Arrowheads indicate the plus end of the microtubule. Scale bar: 5 µm.

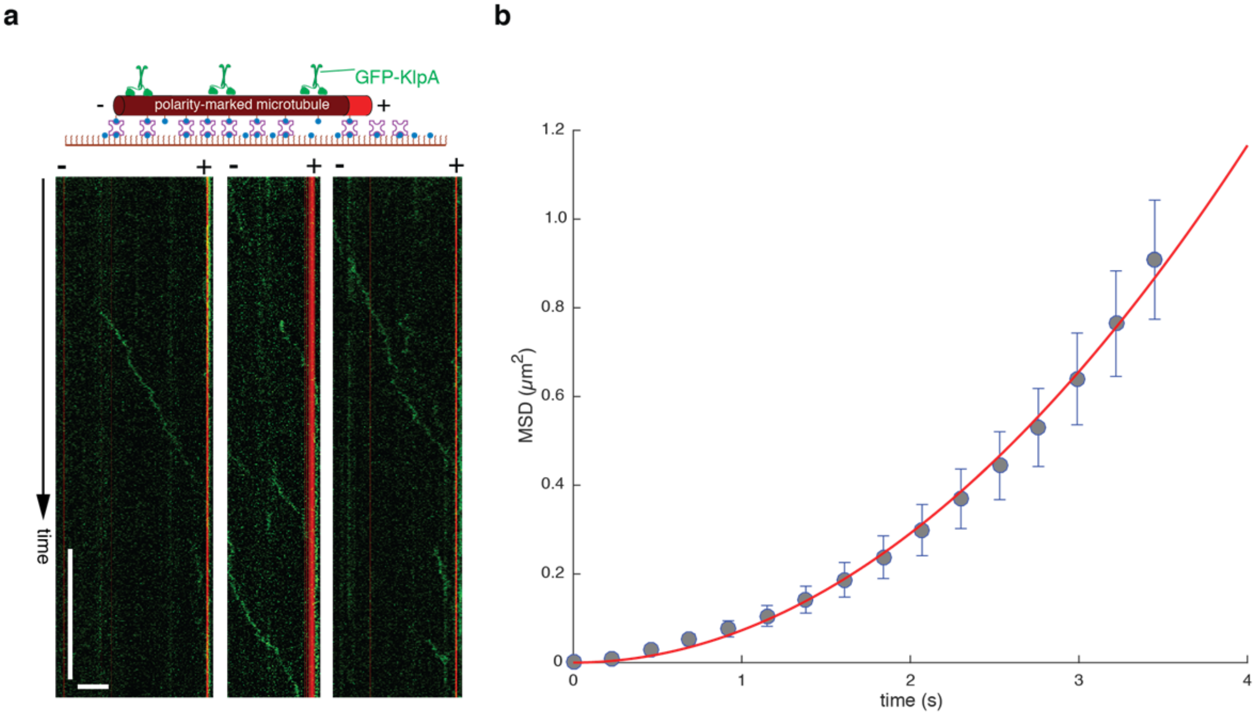


**Supplementary Fig. 3: GFP-KlpA motility is directional on single microtubules. a,** Example kymographs of GFP-KlpA molecules (green) moving in a directional manner on polarity-marked microtubules (red) with a bright plus end and a dim minus end. **b**, MSD analysis of GFP-KlpA on polarity-marked microtubules. The MSD-versus-time plot was best fitted with a one-dimensional directional movement: $MSD = V^2t^2 + offset$. The mean velocity $V$ was determined to be 270 ± 1 nm$^2$ s$^{-1}$ (mean ± s.e.m., n = 79). Scale bars: 30 s (vertical) and 5 μm (horizontal).

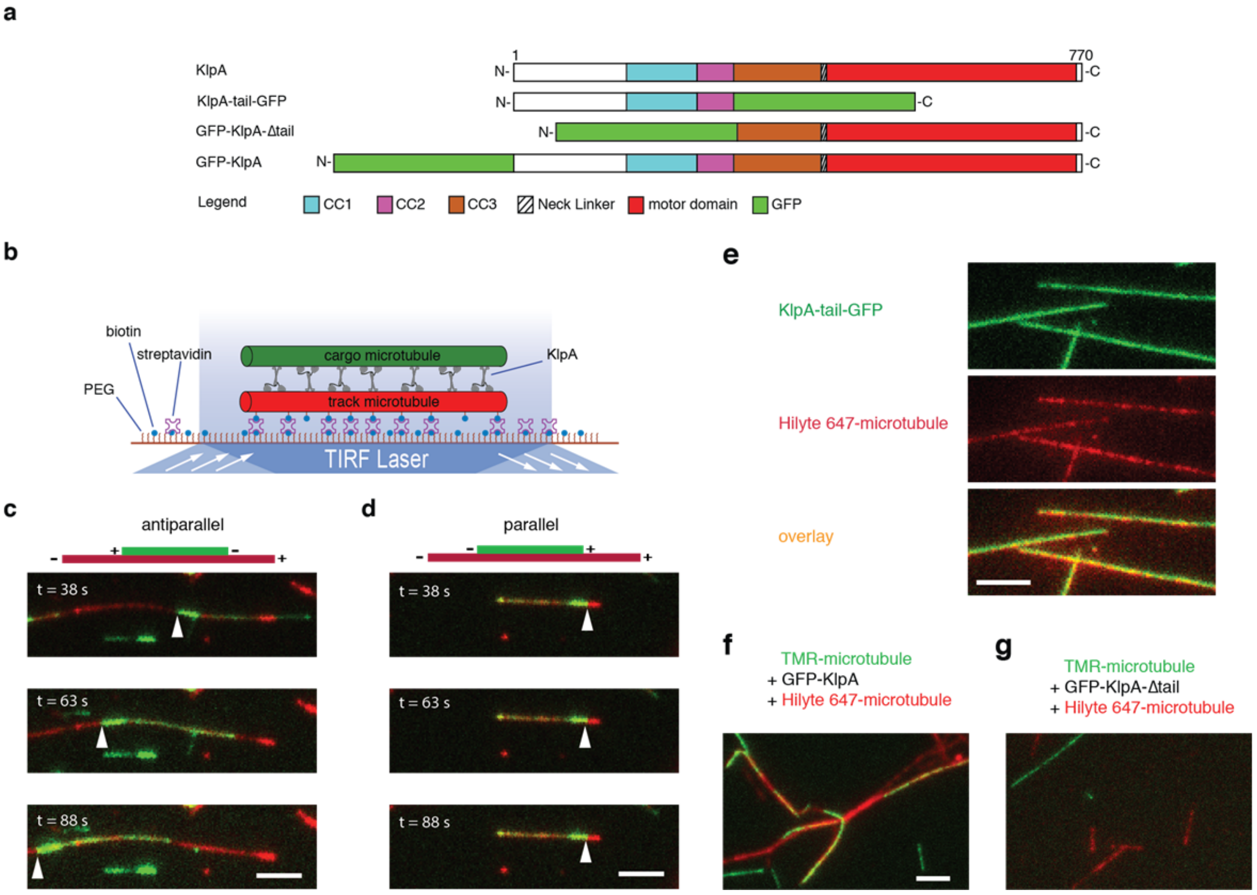


**Supplementary Fig. 4: KlpA slides apart antiparallel microtubules and statically crosslinks parallel microtubules via its N-terminal nonmotor microtubule-binding tail. a,** Schematic diagram of the full-length KlpA, KlpA-tail-GFP, GFP-KlpA-Δtail and GFP-KlpA. KlpA-tail-GFP and GFP-KlpA-Δtail contain residues 1-302 and 303-770 of the full-length KlpA respectively. **b,** Schematic diagram of the microtubule-sliding assay. Polarity-marked track microtubules (red) are immobilized on the coverslip via the biotin/streptavidin/biotin chemistry, and then incubated with KlpA (gray) and polarity-marked cargo microtubules (green). **c, d,** Representative diagrams and image sequences of KlpA dynamically sliding apart antiparallel microtubules in (**c**) and statically crosslinking parallel microtubules in (**d**). Track and cargo microtubules are both polarity-marked with bright plus ends but fluorescently labeled with

different dyes. Arrowheads indicate the plus end of the cargo microtubules. **e,** Microscopy images showing that KlpA-tail-GFP (green) binds to surface-immobilized HyLite 647-microtubules (red). **f, g,** Microscopy images showing that TMR-microtubules (green) readily bundle with HyLite 647-microtubules (red) in the presence of GFP-KlpA (**f**) but not GFP-KlpA-Δtail (**g**). Scale bar: 5 µm.

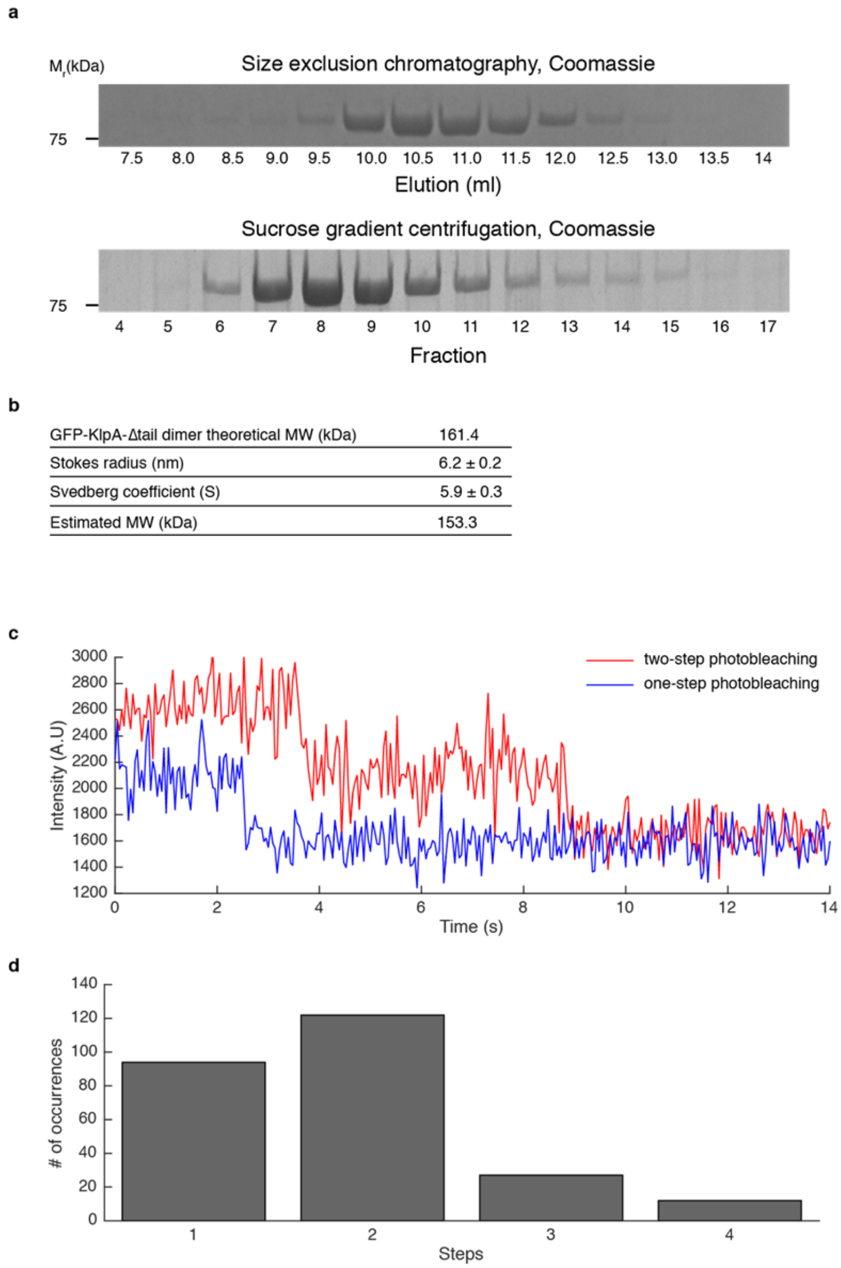

**Supplementary Fig. 5: GFP-KlpA-Δtail forms a homodimer. a,** Hydrodynamic analysis of purified GFP-KlpA-Δtail protein. Fractions from size exclusion chromatography and 5-20% (w $v^{-1}$) sucrose gradient centrifugation experiments. **b,** Calculation of GFP-KlpA-Δtail molecular weight based on the experimentally derived Stokes radius and Svedberg coefficient. **c,** Representative photobleaching traces of GFP-KlpA-Δtail fluorescence on surface-immobilized microtubules in the presence of 1.5 mM AMPPNP. **d,** Histogram of fluorescence photobleaching steps of GFP-KlpA-Δtail (n = 255).

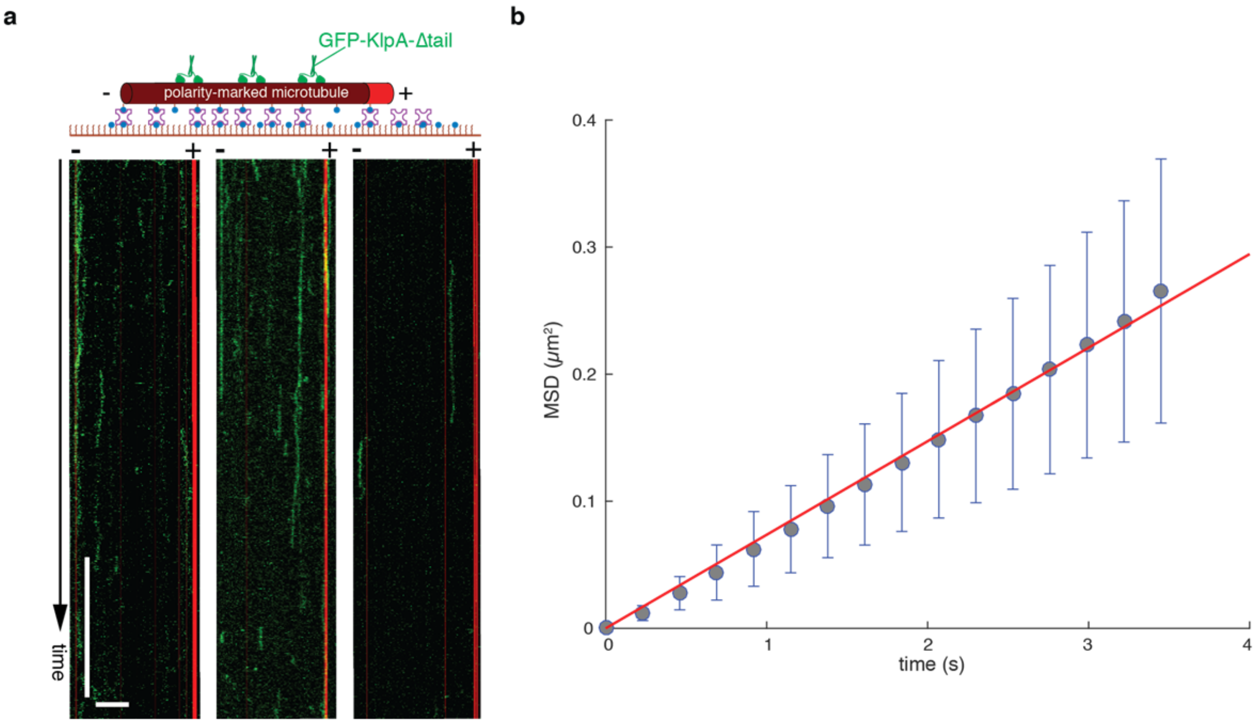


**Supplementary Fig. 6: GFP-KlpA-Δtail is diffusive on single microtubules with no apparent directional preference. a,** Example kymographs of GFP-KlpA-Δtail molecules (green) showing diffusive motility on polarity-marked microtubules (red) with a bright plus end and a dim minus end. **b**, MSD analysis of GFP-KlpA-Δtail on polarity-marked microtubules. The diffusion constant *D* was derived by fitting the MSD-versus-time plot with a one-dimensional

diffusion: $MSD = 2Dt$. The mean diffusion constant $D$ was determined to be 0.0368 ± 0.0003 $\mu m^2\ s^{-1}$ (mean ± s.e.m., n = 40). Scale bars: 30 s (vertical) and 5 µm (horizontal).
